# Particle Deposition in Large-Scale Human Tracheobronchial Airways Predicted by Single-Path Modelling

**DOI:** 10.3390/ijerph20054583

**Published:** 2023-03-04

**Authors:** Cuiyun Ou, Jian Hang, Jiajia Hua, Yuguo Li, Qihong Deng, Bo Zhao, Hong Ling

**Affiliations:** 1 School of Atmospheric Sciences, Sun Yat-sen University, and Southern Marine Science and Engineering Guangdong Laboratory (Zhuhai), Zhuhai 519082, China; 2 China Meteorological Administration Xiong’an Atmospheric Boundary Layer Key Laboratory, Baoding 071800, China; 3 Department of Mechanical Engineering, The University of Hong Kong, Hong Kong, China; 4 College of Public Health, Zhengzhou University, Zhengzhou 450001, China

**Keywords:** computational fluid dynamics (CFD), large-scale human airway, single-path modelling, particle deposition mechanism, deposition prediction

## Abstract

The health effects of particles are directly related to their deposition patterns (deposition site and amount) in human airways. However, estimating the particle trajectory in a large-scale human lung airway model is still a challenge. In this work, a truncated single-path, large-scale human airway model (G3–G10) with a stochastically coupled boundary method were employed to investigate the particle trajectory and the roles of their deposition mechanisms. The deposition patterns of particles with diameters (*dp*) of 1–10 μm are investigated under various inlet Reynolds numbers (*Re* = 100–2000). Inertial impaction, gravitational sedimentation, and combined mechanism were considered. With the increasing airway generations, the deposition of smaller particles (*dp* < 4 μm) increased due to gravitational sedimentation, while that of larger particles decreased due to inertial impaction. The obtained formulas of Stokes number and *Re* can predict the deposition efficiency due to the combined mechanism in the present model, and the prediction can be used to assess the dose-effect of atmospheric aerosols on the human body. Diseases in deeper generations are mainly attributed to the deposition of smaller particles under lower inhalation rates, while diseases at the proximal generations mainly result from the deposition of larger particles under higher inhalation rates.

## 1. Introduction

The adverse health effects of particulate matters (PM) have been studied in epidemiological and toxicological works [1,2,3,4,5]. Previous studies have found that an increased concentration of PM was associated with an increased risk of all-cause and cardiopulmonary mortality, and a higher risk has been found for smaller-sized particles [6,7,8,9,10,11]. There are four main exposure routes for the human body to particulate pollutants, namely ingestion, inhalation, injection and dermal contact [12]. Among them, inhalation is considered as significant because fine airborne particles (particle diameter *dp* ≤ 2.5 μm) can penetrate into the human lung. The novel coronavirus disease (COVID-19) also spreads to susceptible individuals by inhalable droplets or droplet nuclei [13]; therefore, it is essential to know the health effects of particles. Particle deposition patterns, both the rate and the site, in human airways can provide a linkage between PM concentration and health effects [14,15,16].

Computational fluid dynamic (CFD) modelling has been demonstrated to be an effective approach to predict particle deposition in human airways. Most CFD studies have mainly focused on the small-scale modelling of tracheobronchial airways by using single-, double- and triple-bifurcations [17,18,19]. They have found that simple bifurcation models can sufficiently interpret the general airflow structure and particle transport in the certain airways; however, Comer et al. [20] indicated that a simple bifurcation model might not provide a realistic view of particle deposition in the respiratory airways [21]. Accordingly, several studies have developed new methods to model the entire tracheobronchial (TB) airway by integrating the local bifurcation model in parallel and series [22,23,24,25]. However, it is difficult to determine the boundary conditions between local bifurcations, which have significant influences on the prediction of particle transport and the deposition in human airways [23]. Therefore, airway modelling with more generations is necessary.

The direct simulation of particle deposition in multiple-generation airways has become the focus of researchers. van Ertbruggen et al. [26] proposed a three-dimensional airway model in the upper-7-generation airways, and then Gemci et al. [27] studied the airflow in the upper-17-generation airways. However, these models are difficult for wide application due to their consideration of large numbers of branches. Walters and Luke [28] suggested a stochastic coupling method to simulate the airflow pattern and particle deposition in the truncated larger-scale airways (G4 to G12). They found that branches could be partly removed without affecting the deposition simulation in the remaining branches [29]. Tian et al. [30] developed a stochastic individual path (SIP) approach to characterize the transport and deposition of particles throughout the TB region. More applications and validations are warranted for further investigation.

In the present work, we utilized the aforementioned stochastic coupling method. The main idea of this method is to map the static pressure at the resolved airway flow paths to the outlets of the unresolved flow paths in the randomly truncated single-path airway model. Particles with sizes ranging from 1 to 10 μm were computed. Two deposition mechanisms, namely inertial impaction and gravitational sedimentation, were taken into account. Meanwhile, the effects of the particle sizes and airflow rates were evaluated.

## 2. Methods

### 2.1. Truncated Airway Model

**Figure 1:**
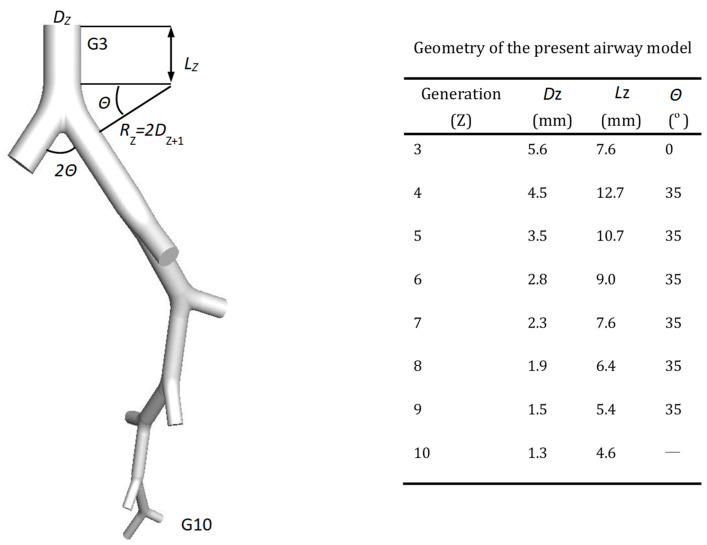
Truncated eight-generation model based on Weibel lung morphology [32].

As obtained in our previous work [31], we could see that the airflow in lower generations (lower than G11) was too small, and the dominant particle-deposition mechanism should be Brownian diffusion, which was not the concern of the present work. Hence, in the present study, an eight-generation airway model (G3–G10) was established based on the data from Weibel [32]. Then, we randomly truncated one of the two daughter branches from the middle downstream of each generation. The truncated branch was named an unresolved outlet, while one of the two branches in G10 remained as the resolved outlet. Hence, there were seven unresolved outlets and one resolved outlet in the model as shown in Figure 1. Parameters of the model are summed in the accompanying table, including the diameter of generation Z (*D_Z_*), the length of generation Z (*L_Z_*) and the angle between 2 daughter branches (2Θ). The curvature radius of the ring connecting the daughter branches (*R_Z_*), which can be calculated by 2*D_Z_*_+1_, is also illustrated in Figure 1. This method is an example of the single flow-path model originally proposed by Tian et al. [30]. To make the truncated geometry faithfully represent the effect of the removed airways, a special boundary condition must be set to the unresolved outlets, which is described in detail in Section 2.3.

### 2.2. Governing Equations

The airflow in the airway model is assumed to be Stokes flow (laminar, steady, and incompressible with constant density and viscosity). The non-dimensional governing equations for the continuity and momentum are given as follows:

(1) ∇·u=0

(2) $$u\cdot \nabla u=-\nabla p+\frac{1}{Re}\nabla^{2}u$$

where *u* and *p* are the dimensionless velocity vector and the pressure, respectively. $u=u^{*}/U$, $p=p^{*}/\rho U^{2}$, where $u^{*}$ and $p^{*}$ are the local velocity and the pressure, respectively; *U* and *D* are the mean velocity at the inlet and the diameter of the inlet, respectively; *ρ* is the air density (1.225 kg/m^3^). The air Reynolds number (*Re*) is defined as Re=ρUD/μ, where μ is the air dynamic viscosity (1.7894 × 10^−5^ kg/(ms)).

The particle phase is assumed to be sufficiently diluted, and subsequently, the transport can be modeled by a one-way coupled Lagrangian method in which the particle motion is described as:

(3) $$\frac{d^{2}x_{p}^{*}}{dt^{2}}=f_{D}\left(u^{*}-u_{p}^{*}\right)+g$$

where $x_{p}^{*}$ and $u_{p}^{*}$ are the particle displacement and the velocity, respectively; g is the gravitational acceleration; $f_{D}$ represents the drag force coefficient per unit particle mass and is defined as:

(4) $$f_{D}=C_{c}C_{D}Re_{p}U/(24S_{tk}D)$$

where $C_{c}$ and $C_{D}$ are the Cunningham correction factor and the drag coefficient [33], respectively. $Re_{p}$ is the particle Reynolds number defined as:

(5) $$Re_{p}=\rho \left|u^{*}-u_{p}^{*}\right|dp/\mu$$

$S_{tk}$ is the Stokes number and is defined as:

(6) $$S_{tk}=\rho_{p}dp^{2}UC_{c}/\left(18\mu D\right)$$

where dp and $\rho_{p}$ are the particle diameter and the density (2000 kg/m^3^), respectively.

### 2.3. Boundary Conditions

Uniform velocity profiles were employed at the inlet. According to the range of oral and/or nasal inhalation airflow rate (*Q* = 5 to 60 L/min, corresponding to the human activities from sleep to intense exercise), the inlet velocity *U* ranged from 0.26 m/s to 5.2 m/s. The corresponding Reynolds numbers ranged from 100 to 2000 in a step of 100. A no-slip velocity boundary condition was applied at all walls. Pressure outlet was employed at the resolved outlets, which was assumed to be equal as atmospheric pressure. We mapped the plane-averaged static pressure at the middle cross-section of the resolved flow-path to the unresolved outlet in the same generation [29], as the airflow was assumed to be symmetrical at the bifurcation. At the beginning of the calculation for the airflow field, we first set the pressure outlet to all the outlets, including both the resolved and unresolved outlets. After iterating several times, the mapping processes were conducted, and then the calculation was completed until the convergence was obtained.

After obtaining the flow field, particles were released uniformly at the inlet. In this work, we injected particles with the same size for each injection. The trap condition was applied to all the walls with the assumption that particles were trapped as soon as they touched the wall surfaces. In other words, the deposition occurred when particles reached the airway wall, and the escape condition was employed for the outlets.

### 2.4. Particle Deposition Efficiency

The regional deposition of particles in human airways can be quantified in terms of the deposition efficiency (*DE*), which is defined as:

(7) $$DE=No_{d}\;/No_{e}$$

where $No_{d}$ is the number of particles deposited; $No_{e}$ is the number of particles entered. We calculated *DE* separately to distinguish the contribution of different deposition mechanisms in the airway model. *DE_i_* corresponds to the inertial impaction. *DE_g_* is the gravitational sedimentation. *DE_i+g_* is contributed by the combined mechanism. Their relationship is described by the following equation [34]:

(8) $$DE_{i+g}=DE_{i}+DE_{g}-DE_{i}\cdot DE_{g}$$

### 2.5. Numerical Methods

The governing equations were numerically solved using a finite volume method by a commercial CFD software ANSYS FLUENT (Version 12.0.16; ANSYS Inc., Canonsburg, PA, USA). The pre-processing tool UNIGRAPHICS (Version 5.0; Siemens PLM Software, Berlin, Germany) was used to build the truncated model, and a structured mesh was generated by GAMBIT (Version 2.4.6; ANSYS Inc., Canonsburg, PA, USA). Refined boundary layers were employed near walls and junctions where the velocity gradient may be larger. The number of final cells for the symmetric geometry was 1,570,000. This number was determined by the grid-independent tests for the flow field solution and particle deposition efficiency, according to the grid convergence index applied by Longest and Vinchurkar [35]. Particles were injected similar to a Dirac function after the steady airflow was achieved, and the disperse phase model was used to calculate the particle trajectories.

The status of particles (e.g., trap, escape, etc.) was recorded once the track of particles was fulfilled. This process was repeated. Then, the average number of particles trapped in specific regions was obtained, which was used for the statistics of the deposition efficiency. Different numbers of released particles (i.e., 10,000, 20,000, 50,000, and 100,000) were considered for the independence tests. No significant change in deposition was found when the number of released particles was larger than 50,000. This method was validated by comparing it with others’ work in our previous paper [35].

## 3. Results

**Figure 2:**
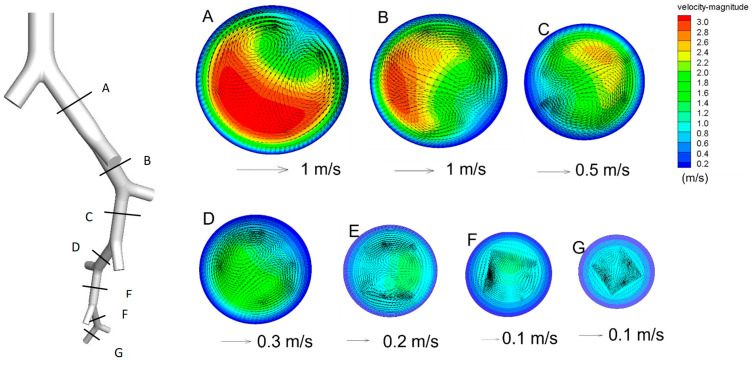
Axial air velocity and secondary airflow vector at various sections (*Re* = 1000).

Figure 2 shows the contours of the axial velocity magnitude and the secondary velocity vector at different cross-sections of different generations when *Re* = 1000 (i.e., an inhalation rate of 30 L/min). Both axial and secondary air velocities were higher near the inner wall of each divider and decreased with each generation. It also shows that the secondary airflow was axisymmetric on the plane of the corresponding divider, and the air moved from the outer side to the inner side in each axis of symmetry, and then produced two symmetric vortices near the axis. The vortices also weakened with each generation.

**Figure 3:**
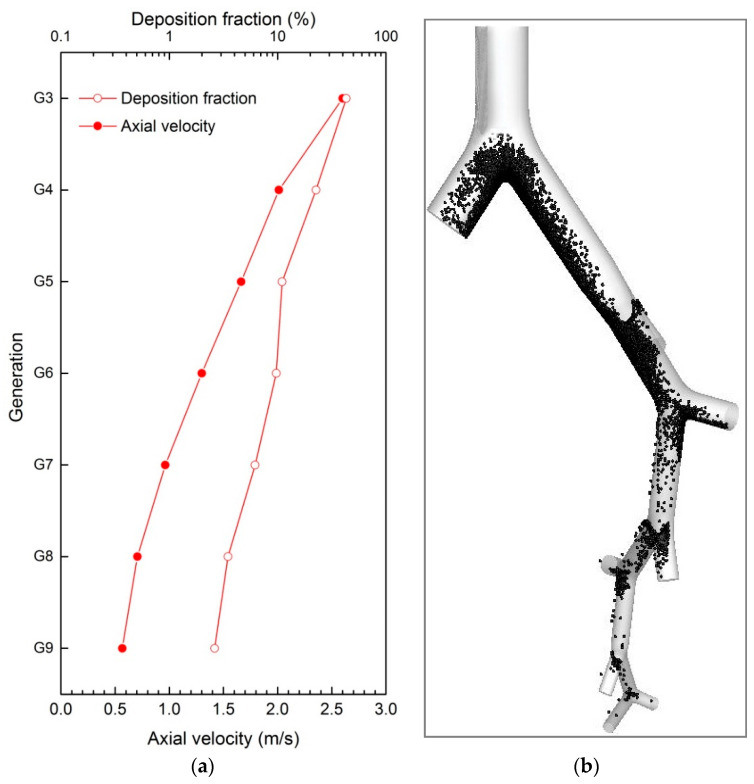
Airflow structure and corresponding particle deposition pattern, including the deposition fraction and deposition sites (*Re* = 1000, *dp* = 10 μm). (**a**) axial velocity and deposition fraction; (**b**) deposition pattern of *dp* = 10 μm.

Figure 3 presents the mean axial air velocity at the beginning section of each generation and the corresponding deposition patterns of 10-μm particles (i.e., *dp* = 10 μm. Particles with other sizes are named following the same way) when Re=1000. The axial air flow rate decreased when the generation number increased, changing from 2.6 m/s (G3) to 0.56 m/s (G9). The corresponding deposition patterns show that 10-μm particles were deposited more in the upper parts than that of in the lower parts. Figure 2B illustrates there are hot spots of deposition for 10-μm particles, particularly at the carinal ridge of each bifurcation.

**Figure 4:**
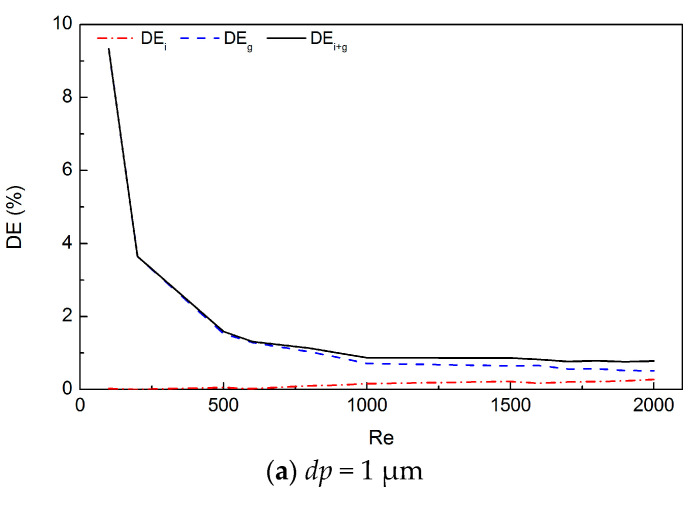

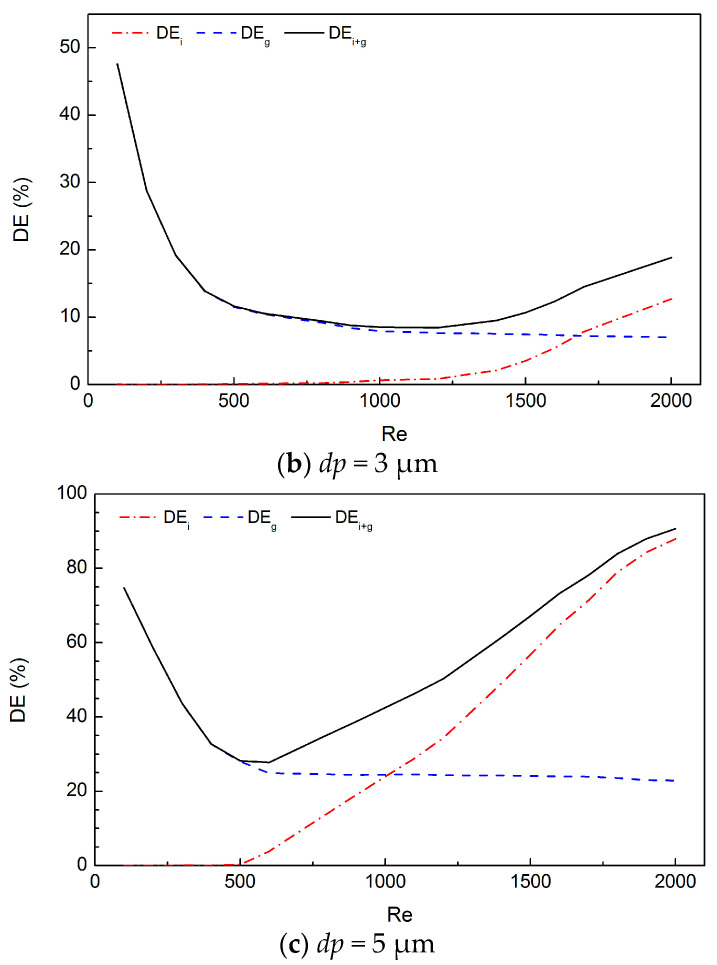
Particle deposition efficiency (*DE*) due to different mechanisms for three different particle diameters (*dp*).

Figure 4 displays the particle deposition in the whole G3–G10 truncated airway model due to different mechanisms for particles of different sizes (1, 3, and 5 μm). In general, the deposition related to inertial impaction (*DE_i_*) increases with rising *Re*, while that ascribed to the gravitational sedimentation (*DE_g_*) decreases. For 1-μm particles, the inertial impaction has few influences on the deposition throughout the entire considered *Re* range. Gravitational sedimentation is dominant when *Re* ranges from 100 to 2000, and the deposition decreases with the increasing *Re*. For 3-μm particles, a U-shape curve of *DE_i+g_* can be found. When Re<800, the gravitational sedimentation was dominant. Hence, *DE_i+g_* decreases rapidly with the increasing *Re*. When Re>1200, inertial impaction prevails, and the *DE_i+g_* curve rapidly rises up with the rising *Re*. When 800<Re<1200, the two mechanisms balance each other, and thus the *DE_i+g_* curve is stable at a low level. For 5-μm particles, the knee point (when gravitational sedimentation balances inertial impaction) of the *DE_i+g_* curve moves from higher *Re* to lower *Re,* compared to that of 3-μm particles.

**Figure 5:**
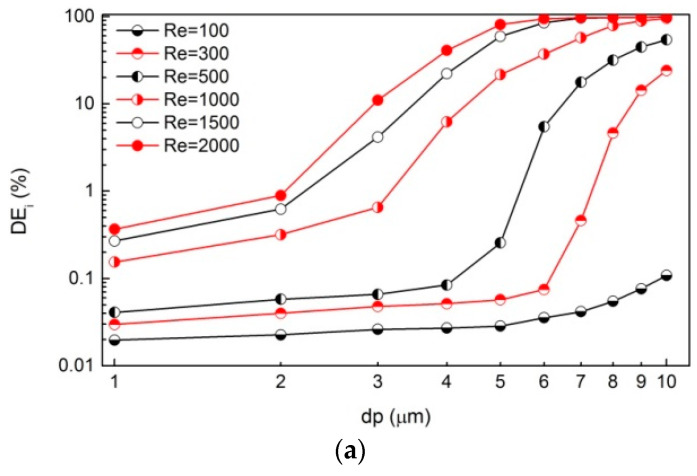

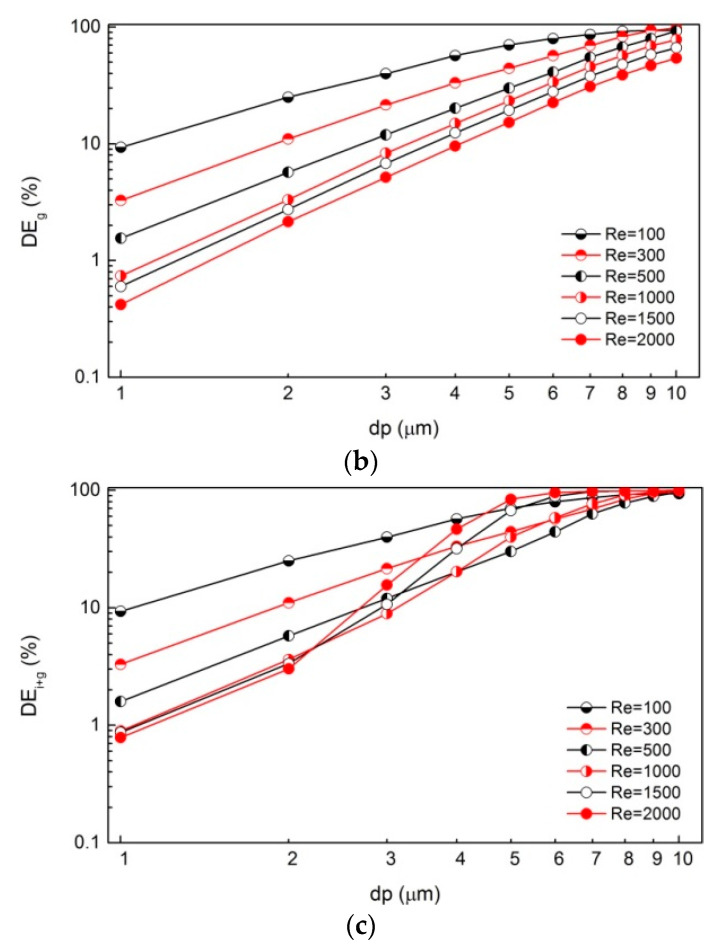
Particle deposition efficiency (*DE*) vs. particle diameter (*dp*) due to different mechanisms, including (**a**) inertial impaction; (**b**) gravitational sedimentation; (**c**) combined mechanism.

Figure 5 shows curves of *DE* with particle diameter (*dp*) ranging from 1 to 10 μm in logarithmic coordinates. Generally, depositions dominated by all three mechanisms are enhanced with the increasing *dp*. For the inertial impaction, *DE_i_* increases slowly below a critical *dp* (e.g., dp=5 μm when Re=100), while it increases rapidly after crossing the knee point. The larger the *Re* is, the smaller the critical *dp* is. For the gravitational sedimentation, *DE_g_* increases with the rising *dp* (i.e., a linear increase in the logarithmic coordinates). For the combined mechanism, *DE_i+g_* curves display similar trends as *DE_g_* curves when Re≤500. When Re>500, the variation in *DE_i+g_* is more similar to that of *DE_i_*, only the critical *dp* is slightly changed for each *Re*.

**Figure 6:**
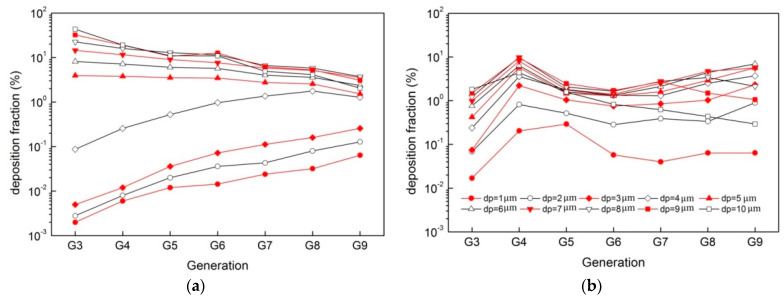
Generational deposition fractions for particles in multi-size (*dp* = 1 µm to 10 µm) when *Re* = 1000, (**a**) inertial impaction; (**b**) gravitational sedimentation.

Figure 6 depicts the deposition fraction in different generations (G3–G10). The deposition fraction due to inertial impaction increases with generations for small particles (dp≤4 μm), while it decreases for larger particles (dp≥5 μm). The deposition fraction due to gravitational sedimentation increases slowly from G3 to G9 (except the relatively high deposition fraction at G4) for particles from 1 to 7 μm but decreases if *dp* = 8–10 μm.

**Figure 7:**
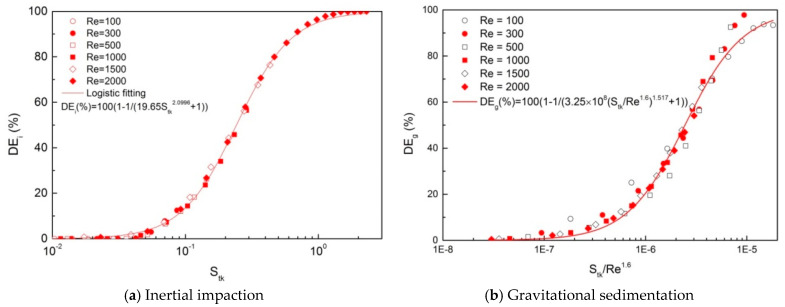
Variation of particle deposition efficiency (*DE*) in terms of non-dimensional parameters: (**a**) *S_tk_* for inertial impaction and (**b**) *S_tk_*/*Re*^1.6^ for gravitational sedimentation.

Figure 7 shows the “S” shape variation curves of *DE* in terms of non-dimensional parameters. Figure 7a presents the growth curves of *DE_i_* that associated with *S_tk_* only. The fitting curve can be represented by a logistic regression as following:

(9) $$DE_{i}\;\left(\%\right)=100\left(1-1/(19.65S_{tk}{}^{2.0996}+1\right))$$

Figure 7b illustrates the variation in *DE_g_* in terms of $S_{tk}$/$Re^{1.6}$. It can be fitted to a logistic regression curve such as the following:

(10) $$DE_{g}\;\left(\%\right)=100\left(1-1/(3.25\times 10^{8}{\left(S_{tk}/Re^{1.6}\right)}^{1.517}+1)\right)$$

## 4. Discussion

This study predicted the fate of particles and the deposition mechanisms in a large-scale TB airway model. We first assessed the influences of the airflow and respiratory parameters on the particle deposition patterns. We then analyzed the factors that affected the *DE* by using three different mechanisms: inertial impaction, gravitational sedimentation, and a combined mechanism. Finally, we investigated the generational deposition fractions resulting from the mechanisms. We established that the trajectories of particles in the human lung can be influenced by the airflow structure, *dp*, and deposition mechanism. Furthermore, we obtained a formula in terms of *S_tk_* and *Re* to predict the deposition efficiency (*DE*) due to the combined mechanism in the present model. Finally, we gave a suggestion for assessing the dose-effect of surrounding particulate pollutants on the human body.

We used the method of three-dimensional (3-D) truncated single-path airway model. Gemci et al. [27] modelled the whole computer-tomography-based TB (G0–G16) airway including 1453 bronchi, although they truncated nearly 99% of the bronchi to simplify the computation. They determined the outflow boundary conditions at outlets by using mass distributions, leading the predicting pressure drop approximately 30% lower than the experimental results obtained by Hyatt and Wilcox [36]. To reduce the computational expenses effectively, numerous researchers have put their efforts into developing new methods to model the airflow and the particle transport patterns in the large-scale human lung airway model. Walters and Luke [28] proposed a method that stochastically mapped the plane-averaged static pressure at the middle cross-section from one of the resolved flow paths to the unresolved outlets in the same generation in a symmetrical G4–G12 airway model. The stochastically individual path (SIP) airway modelling approach suggested by Tian et al. [30] considered the influence of different lung lobes on boundary conditions at upper generations of TB airways. Different ventilation modes in the five lung lobes were used to determine the flow rate at the corresponding bronchi for outflow boundary conditions. Tena et al. [37] proposed a method that applied velocities mapped to the unresolved sections from similar locations in fully resolved airway sections to model the lung airway up to G16. The approaches proposed by Walters and Luke [28] and Tian et al. [30] were efficient in simplifying the airway geometry for modelling particle deposition patterns in large-scale human lung airways; however, there are insufficient applications or validations. Our previous study [35] adopted the method of Tian et al. [28] to compare the particle deposition patterns of human and ferret TB airway models (G0–G16). We found that this method could effectively complete such a large-scale airway simulation; therefore, the present work adopted this method to simulate the particle deposition in the single-path G3–G10 airway model that can be considered as one conducting airway of the five lung lobes.

We found that the airflow structures can influence the particle deposition, which is consistent with previous studies [38,39,40]. For example, Comer et al. [41] found that the three-dimensional airflow influenced the particle deposition in a double-bifurcation model. Zhang and Kleinstreuer [42] indicated that the particle deposition pattern in a triple-bifurcation model was highly influenced by the airflow structure. Rahimi-Gorji et al. [43] found that the maximum velocity change occurred in the larynx might cause more particle deposition at this location. Likewise, in our G3–G10 model we found the particle deposition pattern was greatly affected by the airflow structure. When the airflow velocity decreases with the rising generation, the generational deposition fraction of smaller particles (e.g., *dp* = 4 μm) increases, however, that of larger particles (e.g., *dp* = 10 μm) decreases.

We found that inertial impaction and gravitational sedimentation play important roles in the deposition fraction at different sites of the airway. Inertial impaction leads the increased deposition fraction of smaller particles (1 μm ≤ *dp* ≤ 4 μm) and the decreased deposition fraction of larger particles (5 μm ≤ *dp* ≤ 10 μm) with the rising generation. Gravitational sedimentation dominates the deposition of smaller particles but not for larger ones. Thus, the gravity only works on smaller particles depositing at lower generations. Therefore, larger particles mainly deposit at upper generations due to inertial impaction, while smaller ones are mainly concentrated at lower generations due to gravitational sedimentation. We also noticed an exception to this rule that a relatively high deposition fraction was observed at G4 due to gravity for particles in the entire size range. This exception may be influenced by the inlet boundary [21] as well as the morphological configuration (i.e., the bifurcation orientation [20], the lengths and the diameters of each generation tube [44], and the geometry parameters of each bifurcation divider [45]). The model applied in this work can be easily extended to the whole TB airway; hence, it can be used to precisely derive the particle trajectories in the lung and can help better understand the health effect of PM.

The strength of our study lies in the fact that we separated the roles for different deposition mechanisms in large-scale human lung TB airways. The deposition mechanism is one of the physical factors that determine the particle deposition pattern in the human respiratory tract [46]. Particle deposition in the lung airways mainly occurs owing to three mechanisms: inertial impaction, gravitational sedimentation, and Brownian diffusion [47]. Previous studies have focused on either the deposition mechanisms in simple bifurcation airway models [48,49,50] or the deposition patterns in complicated airway models but without precise descriptions for the mechanisms [51,52], which cannot shed light on the deposition mechanisms in depth. There are studies of gravitational deposition that have concentrated on the small bronchial airways and alveolar region. Sedimentation has been found playing the dominant role in these regions [53,54]. In our work, we investigated the variation in *DE_i_*, *DE_g_*, and *DE_i+g_* in terms of *Re* and *dp*. The sedimentation was found dominating the inertial deposition under small-*Re* conditions, while the inertial impaction become dominant over sedimentation with the increasing *Re*. *DE_i_*, *DE_g_*, and *DE_i+g_* were found increasing with *dp*. The findings are in good agreement with many previous studies, indicating that particle deposition in the TB region increases with *dp* rising from 1 to 10 μm [47,55,56].

Another merit of this study is that we established the relationships between non-dimensional parameters and *DE_i_*, *DE_g_*, and *DE_i+g_*, respectively. First, we found that *DE_i_* varied with $S_{tk}$ in a consistent regression curve for the entire range of inlet *Re* and obtained a statistical regression equation of $S_{tk}$. Then, we found that *DE_g_* varied with $S_{tk}$/$Re^{1.6}$ in a consistent way. Finally, the contribution of the combined mechanism increased with the particle size; however, it decreased with *Re* when Re≤500 and increased with *Re* when Re≥1000. Therefore, we failed to find a single parameter to characterize *DE_i+g_* throughout the whole range of *Re* and the particle size. Nevertheless, we can describe it separately using $S_{tk}$ (when Re≤500) or $S_{tk}$/$Re^{1.6}$ (when Re≥1000). Cheng et al. [57] found that oropharyngeal deposition (equivalent to *DE_i+g_* in the present work) had a good correlation with $S_{tk}$ only, while the Reynolds number had no effect on deposition for flow rates ranging from 15 to 60 L/min under which conditions of inertial impaction dominated over gravity. Kleinstreuer et al. [48] suggested that *DE* could be correlated only with $S_{tk}$ under normal breathing conditions (i.e., *Q* ≥ 15 L/min), while under slow breathing conditions (i.e., *Q* ≤ 7.5 L/min), sedimentation became increasingly dominant over inertial impaction. This finding agrees well with our present results. Chen et al. [17] suggested that deposition in the airway model of generations G11–G14 were determined by $S_{tk}$ and the sedimentation parameter (which is also a function of airflow rate) for particles in the size range of 1–7 μm. This conclusion also agrees well with our present results.

The third merit of our study is that we probably have provided a formula in terms of Stokes number ($S_{tk}$) and Reynolds number (*Re*) to predict the deposition efficiency due to the combined mechanism (*DE_i+g_*). We first obtained the equation of *DE_i+g_* by replacing the formula (9) and formula (10) into formula (8) as follows:

(11) $$DE_{i+g}\left(\%\right)=100-100/\left(\left(19.65S_{tk}^{2.0996}+1\right)\left(3.25\times 10^{8}{\left(S_{tk}/Re^{1.6}\right)}^{1.517}+1\right)\right)$$

**Figure 8:**
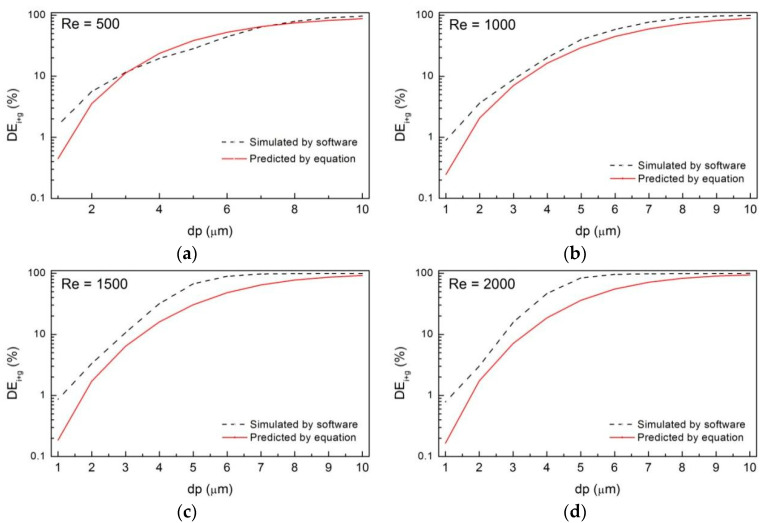
Comparisons of the values of *DE_i+g_* between the prediction and simulation, including (**a**) *Re* = 500; (**b**) *Re* = 1000; (**c**) *Re* = 1500; (**d**) *Re* = 2000.

Then, we calculated the value of *DE_i+g_* for particles in the size range of 1–10 μm under different *Re*, namely values predicted by equation. Finally, the comparisons of the values between those predicted by equation and those simulated numerically are displayed in Figure 8. From this figure, we can find that the accuracy of the prediction decreases with *Re* but increases with particle size, which can be obviously found by calculating the relative error between prediction value and simulation value, as shown in Table 1. When *Re* = 500, the relative error ranges from −71% to 36% with a mean value of −21%, and the maximum value occurs when *dp* = 1 μm, while the minimum occurs when *dp* = 7 μm. When *Re* = 2000, the relative error ranges from −79% to −6% with a mean value of −40%, and the maximum value occurs when *dp* = 1 μm, while the minimum occurs when *dp* = 10 μm. In other words, the prediction of the deposition efficiency in the large-scale airway model can assess the dose-effect of surrounding particles to human body. The larger the particles are, or the lower the inhalation rates are, the better the accuracy of the prediction can be.

Our model can be further improved, as it is a symmetrical airway model, while a realistic human airway should be asymmetrical, and asymmetry also affects the particle deposition [58,59]. The asymmetric geometries, such as different lobes, will lead to heterogeneous ventilation in different lung regions due to the nature of each region volume. A realistic, fluctuating inlet velocity profile should be considered in the future work because the inlet airflow patterns also affect the particle deposition [60,61,62,63]. The orientation of the gravity may play an important role in the deposition due to sedimentation, while the present work only takes into account the orientation perpendicular to the inlet of the model. Moreover, the particle release pattern also affects the particle deposition pattern [44,64], which certainly warrants further study.

Furthermore, this work is essential for a better understanding of the correlations between human health and characteristics of aerosols (e.g., concentration distribution, dispersion, physical and chemical composition, toxicology, etc.). In our future work, we plan to couple the current work with an atmospheric dispersion model and an exposure model, connecting the research of inhalable aerosols with in vivo deposition, exposure, and morbidity of respiratory diseases. Using the current work as a crucial tool, a series of the interdisciplinary studies of air pollution and public health are planned.

## 5. Conclusions

This work uses a truncated single-path large-scale human airway model (G3–G10) and a stochastically coupled boundary method for investigating the particle trajectory and the roles of deposition mechanisms in the human lung airway. Particle size ranging from 1 to 10 μm, various inlet Reynolds numbers (*Re* = 100–2000), and 3 deposition mechanisms (inertial deposition, gravitational sedimentation, and combined mechanism) are considered in this research. We found the truncated single-path lung model efficiently estimates the fate of particles in the lung. Particle size and inhalation rate were found to be key factors that determine the deposition site, amount, and mechanism. A formula is obtained in this work for predicting the deposition efficiency due to the combined mechanism (*DE_i+g_*), using non-dimensional parameters, Stokes number (*S_tk_*), and Reynolds number (*Re*). Our findings indicate that the prediction of the deposition efficiency in the large-scale airway model can assess the dose-effect of surrounding particulate pollutants to human body. The larger the particles are, and/or the lower the inhalation rates are, the better accuracy the prediction can be. Our results imply that diseases that occur in deeper generations are mainly due to the deposition of smaller particles under lower inhalation rates. Meanwhile, diseases that occur in the proximal generations mainly result from the deposition of larger particles under higher inhalation rates.

## Figures and Tables

**Table 1:** The relative error between the prediction value and simulation value.

*dp* (μm)	*Re =* 500	*Re =* 1000	*Re =* 1500	*Re =* 2000
1	(0.72)	(0.72)	(0.79)	(0.79)
2	(0.36)	(0.42)	(0.49)	(0.43)
3	(0.01)	(0.19)	(0.41)	(0.55)
4	0.22	(0.20)	(0.50)	(0.61)
5	0.37	(0.26)	(0.55)	(0.57)
6	0.19	(0.23)	(0.45)	(0.42)
7	0.02	(0.22)	(0.34)	(0.28)
8	(0.06)	(0.20)	(0.23)	(0.16)
9	(0.10)	(0.14)	(0.13)	(0.10)
10	(0.10)	(0.10)	(0.07)	(0.06)
Mean relative error	(0.21)	(0.27)	(0.40)	(0.40)

Note: Values in parenthesis are negative values, which means underprediction.

## Data Availability

The data presented in this study are available in the figures of this paper.
